# Media awards for responsible reporting of suicide: Experiences from Australia, Belgium and Denmark

**DOI:** 10.1186/1752-4458-5-15

**Published:** 2011-06-03

**Authors:** Andrew J Dare, Karl AM Andriessen, Merete Nordentoft, Michella Meier, Annemiek Huisman, Jane E Pirkis

**Affiliations:** 1 Melbourne School of Population Health, The University of Melbourne, Melbourne, Australia; 2 Suicide Prevention Program of the Flemish Mental Health Centres, Gent, Belgium; 3 Flemish Working Group on Suicide Survivors 'Verder', Gent, Belgium; 4 Psychiatric Centre Copenhagen, Mental Health Services in the Capital Region, Copenhagen University, Denmark; 5 Department of Clinical Psychology, Vrije Universiteit Amsterdam, Amsterdam, The Netherlands

## Abstract

**Background:**

Media awards to encourage responsible reporting of suicide have been introduced in several countries, including Australia, Belgium and Denmark.

**Aims:**

This study aimed to examine the experiences of Australian, Belgian and Danish award recipients in preparing stories on suicide, and consider the impacts of the awards for these recipients and for media professionals more broadly.

**Method:**

We conducted semi-structured telephone interviews with the majority (14 out of 15) of past recipients of the awards in the three countries of interest.

**Results:**

Media awards appear to show promise as a method of reinforcing national and international media guidelines on reporting suicide. The recipients of awards were proud to have had their achievements recognized in this way, and had developed a heightened awareness of the issues inherent in reporting suicide. Although relatively few had prepared subsequent stories on suicide, a number had been given opportunities to provide advice to other media professionals about how best to approach this sensitive topic. Recipients viewed the awards as an important means by which good quality reporting can be rewarded, and a springboard for raising community awareness about suicide.

**Conclusion:**

The experience from Australia, Belgium and Denmark suggests that media awards which recognize responsible reporting of suicide are extremely worthwhile.

## Introduction

Research has consistently demonstrated that media reporting of suicide can lead to 'spikes' in suicide rates than cannot be explained by other factors [1,2]. Phillips named this phenomenon the 'Werther effect' in reference to the spate of copycat suicides in Europe that followed the release of a romantic tragedy by Goethe in which the protagonist took his own life [3].

The main response to this has been the development and dissemination of guidelines to encourage responsible reporting among media professionals. The World Health Organization (WHO) and the International Association for Suicide Prevention (IASP) have developed international guidelines [4], and government and non-government bodies in many countries have developed national guidelines [5]. These guidelines typically caution media professionals against sensationalizing suicide, giving it undue prominence, and providing explicit details about suicide methods. They are not about censorship, however, and in fact acknowledge the important role the media has in dispelling myths about suicide and providing information about where vulnerable readers or viewers might seek help [5]. Evaluative evidence is mounting that this approach can improve the quality of journalism in this area [6-11].

The production of guidelines is beginning to be complemented by other proactive incentives to encourage responsible reporting. In particular, several countries - including Australia, Belgium and Denmark - are now offering awards which honour media professionals who report suicide-related issues in an exemplary fashion in print or broadcast media:

Australia's awards have been in place since 2004, and are auspiced by *Suicide Prevention Australia*. The awards recognise media organizations or individuals who 'have assisted or provided effective and accurate delivery of information on suicide and suicide prevention and through that reporting assisted in progressing awareness and suicide prevention' [12]. The awards are assessed by a judging panel, chaired by a Suicide Prevention Australia Board Member, and consisting of representatives from a range of suicide prevention, mental health and media organisations. The awardees are announced on World Suicide Prevention Day each year. The awards complement Australia's national guidelines on reporting suicide, *Reporting Suicide and Mental Illness *which were developed by the Department of Health and Ageing [13] and have been disseminated by the Hunter Institute of Mental Health [14].

The Belgian *Award for Responsible Portrayal of Suicide and Survivors *is auspiced by the Flemish Working Group on Suicide Survivors, which was also responsible for developing and disseminating guidelines on the reporting of suicide, *Als Journalist Kan Je Levens Redden [As a Journalist You Can Save Lives] *[15]. The award was launched in 2003 as a part of the Working Group media policy, and has been offered since 2004 [16]. Each year a jury of five members is appointed by the Flemish Working Group on Suicide Survivors, with a suicide survivor as Chair. Other members include a media representative and a suicidologist. Following installation of the jury, the Flemish Working Group on Suicide Survivors makes a public call for nominations, via a press release and announcements on its website and in its monthly e-newsletter. The jury considers nominations against the guidelines on reporting and agrees on a winner. Awards are presented on Suicide Survivor Day, in November of each year. The award and the dissemination of the guidelines were included in the Flemish governmental Suicide Prevention Action Plan 2006-2010.

In Denmark, the auspicing body for the annual *Werther Award *is the Danish Association for Education and Research in Suicide Prevention. The award has been offered since 2005, and is judged by the 'Werther Committee' which comprises representatives from the Danish Association for Education and Research in Suicide Prevention, the Centre for Suicide Research, the Danish Journalists' Union and the Association for Survivors after Suicide. Nominees are invited via a press release, and the committee selects awardees based on nominations received. The winner is announced on World Suicide Prevention Day. The Werther Award promotes the principles of responsible reporting of suicide outlined in the WHO/IASP guidelines, but Denmark does not have its own national guidelines.

To date, there has been no formal exploration of the benefits of such awards. The current study examined the experiences of Australian, Belgian and Danish award recipients in preparing stories on suicide, and considered the impacts of the awards for these recipients and for media professionals more broadly.

## Method

We approached the relevant auspicing bodies in Australia, Belgium and Denmark and asked them to provide us with lists of previous winners of the awards, and a summary of the stories for which the awards were granted.

We then contacted the winners and a member of our team conducted a semi-structured interview with each of them. During these interviews, the winners were asked to respond to a set of questions. Most of these questions were closed-ended, but, depending on the winner's response, the interviewer probed further to explore his or her experiences in more detail. The questions were:

1. You were presented with an award for your story on *[describe story]*. When you prepared this story, what was your aim?

2. When you prepared this story, were you aware of any guidelines on reporting suicide?

3. Since winning the award, have you prepared any other stories on suicide?

4. Did winning the award make you reflect on how others report on suicide?

5. Has winning the award resulted in your giving advice to others on how to report on suicide?

6. Has winning the award had any other impacts for you?

In Australia and Denmark, all interviews were conducted via telephone, recorded, and transcribed. In Belgium, all but one of interviews were also conducted by telephone and recorded and transcribed, and one was conducted via email. The Australian interviews were conducted and transcribed in English, and the Belgian and Danish interviews were conducted in each country's main language and then translated into English following the transcription phase.

English transcripts from all three countries were analysed by the primary author (AD), in order to explore prominent themes emerging from the interviews. These themes were cross-checked with the authors with overarching responsibility for the Belgian and Danish interviews (KA and MN, respectively) in order to ensure accuracy of interpretation.

## Results

In total, we conducted interviews with 14 award recipients of a potential 15: five from Australia (four who had prepared television stories and one who had prepared a newspaper story; one was not available due to unforeseen circumstances); five from Belgium (two who had prepared newspaper stories; two who had prepared stories for magazines; and one who had written a book); and four from Denmark (two who had prepared television stories; one who had prepared a newspaper story; and one who had written a book).

### Aims of award-winning stories

Award recipients from the three countries noted that their intention when preparing the winning story was to raise awareness of the risk factors surrounding suicide. Some of these risk factors were common across countries, such as depression. Others were more localized, such as drought and other pressures associated with living in rural and remote areas (mentioned by Australian recipients).

Awardees in each country noted that they hoped that their story might help to reduce the stigma associated with suicide. Some also mentioned a desire to alert loved ones to 'signs' of suicide risk, and to 'tell a story' that would encourage the audience to empathise with those bereaved by suicide.

### Awareness of guidelines on reporting suicide

Australian participants reported a prior awareness of the issues related to responsible reporting of suicide-related issues, although they noted that this awareness came from not only from the national guidelines but also from the codes of practice of their own organizations. Only one participant had not been previously aware of these issues but, once assigned the story, was quickly directed to Australia's national guidelines by a colleague, suggesting some broader awareness of issues related to responsible reporting of suicide in that organisation, at least::

'From memory I think it was another fellow reporter from [other television station] and she had, because I think she'd done this course or something and she said ... she just lent me her manual and I think it came with a CD and stuff, which would be really helpful for you when you are doing the story, so I don't think it was something that my immediate managers were aware of, I am not sure, I can't remember.'

The Belgian awardees also reported an awareness of the existence of guidelines for responsible reporting of suicide prior to their receipt of the award, with some mentioning the recommendations from the Flemish Working Group on Suicide Survivors and others mentioning the WHO/IASP guidelines. Several noted that their awareness came from previous experience in reporting suicide-related issues:

'I did know the recommendations, and this was already my third book on suicide. I knew the recommendations from the Flemish Working Group on Suicide Survivors. Besides, I am a survivor and I'm aware of the sensitivity needed to talk about suicide, without taboo and straightforward.'

All four of the Danish award winners reported that they were familiar of the WHO/IASP guidelines prior to being nominated and winning an award. Again, several noted that this familiarity came from having previously reported on suicide:

'I knew about WHO's guidelines and I considered them before I wrote, but I have written about suicide both before and after winning the award, so it wasn't a new territory.'

One Danish awardee stated although she was aware of the WHO guidelines, she and her co-producers used recommendations from their own local codes of practice to prepare suicide-related news material:

*'I believe that WHO has some very good guidelines, but I wouldn't say that we followed them as our guidelines. At *Denmark Radio *[Denmark's government-funded television and radio broadcasting organisation] we have some very good guidelines on how to approach a lot of topics, amongst those suicides, and those were the ones we used.'*

### Preparation of subsequent stories on suicide

Only one of the award recipients had prepared further suicide-related stories, although several others indicated that they anticipated that they would in the future and would be enthusiastic about the opportunity to do it well. Those who had not prepared subsequent stories indicated that this was not due to any lack of interest on their part, but rather the broader agendas of news media reporting.

In Belgium and Denmark, the situation was similar. Again, only one award recipient from each country had prepared a further story on suicide, largely because of competing imperatives at their media outlets. The remainder indicated that they would willingly do so in future, should the opportunity arise.

### Reflections on how others report on suicide

A minority of Australian winners reported that the award had no effect on their awareness of how other media professionals report on suicide. The majority, however, indicated that it had given them new insights into their peers' reporting practices. The responses of two award recipients illustrate this point:

'Yes, yes it does... I am very aware when people trivialise issues of suicide issues and attempts at the same time I am aware of people over-dramatising it, and that's where I think we go most of the time, thinking it's somehow cool and trendy, and so I was very conscious when we were cutting it that we were going to be sort of bold about it in the sense it's a nuts and bolts fact of life that a lot of people have to deal with these issues but we are not going to romanticise it nor are we going to dramatise it, nor are we going to stigmatise it, the three are just as bad really.'

'It gave me increased scope to see what other journalists are doing with stories related to suicide and mental illness. It makes you more aware of the different approaches others take to report these kinds of stories.'

Belgian award recipients asserted that winning the award had raised their awareness of how others report on suicide in their own organisations. They noted that there are sometimes competing news production imperatives which make it difficult to comply with recommendations in the guidelines. One award recipient made the following comment:

'It happens that a discussion occurs at the editor's desk regarding how to bring a certain story. Usually, suicide is not reported as a news item, however, sometimes it happens when it concerns an equivocal situation, for example when a missing person is found dead. In general, we try to comply with the media guidelines but we don't succeed always, for example sometimes there is no telephone number below an article, or sometimes the suicide method was mentioned.'

Danish award recipients commented that winning the award had made them more alert to examples of poor quality reporting, such as insensitive stories, or stories that provide explicit information about the method of suicide.

'Not long ago there was a news story in the media about a young person who committed suicide which was quite similar to the story my partner and I made. I noticed that it was being written about in the media in a very untactful manner and that they didn't hold back when writing about all the grim details. That incident really made me think about the ethical aspect when writing about suicide.'

### Provision of advice to peers

Australian award winners had mixed responses on this topic, largely related to the extent to which opportunities to provide advice to peers had arisen for them. Most indicated that they would have been happy to share information about responsible reporting of suicide but that their advice had not been sought. Others said that they had either volunteered information or been actively approached by local and international peers for advice regarding responsible reporting on suicide. In these cases, the given award recipients provided advice but noted that it was not always acted upon. The following comments exemplify these responses:

'It has given me increased incentive to speak to colleagues about what I have learnt in my process of reporting these kinds of stories, especially in terms of advice to them about things such as appropriate language, for example referring to someone who has suicided as "an angel", which gives the wrong impression as suicide being a positive solution to problems.'

'It did make me reflect on that and interestingly enough after I did that story I had a call from [an overseas television station] and they wanted to use my audio from my stories or they wanted me to put them in touch with [the community that was the focus of the original story] because they saw my story and they wanted to do something on [the same community] and then you know I put them in touch you know with the community but they didn't use my story, they ended up interviewing someone from the community about it and the reporter at the time sent me an email and said here's the link, this is the interview if you are interested in hearing it, so I listened to the interview and she'd actually talked about how they committed suicide and all that, so it was quite, because I was being so careful with my story and then I heard her interview and I sort of probably shouldn't have but it just wrote back and said "do you guys have mental health guidelines over there", and she wrote and said "yes we do", and then that was that, I can't really tell anyone else what they need to do... that was the actual broadcast, because she put it on the website, because it was on their website and she just sent me the link to the website and so it was on there and it was just like... I can't remember how he committed suicide but they actually explained you know how it was done and stuff, I was really disappointed that I recommended people and I just assumed that they were going to be like me.'

The Belgian participants in this study also reported mixed experiences with providing advice to colleagues following their receipt of the award, again because the opportunity had arisen for some and not for others. All indicated a willingness to share their knowledge:

'That hasn't happened yet, but I surely would do it if there was an opportunity.'

'Yes, to my colleagues of the news editorial desks of the public broadcasting company. After receiving the media award I (have) forwarded the media recommendations five times... Usually I received a kind of amazed reply to my posting. I asked the news editors to include background information, help resources for the readers, and to use appropriate wordings.'

Like their Australian and Belgian counterparts, the Danish award recipients reported varying opportunities to provide advice to their professional peers about appropriate ways of reporting suicide. Where award recipients had provided advice to peers, they had clearly done so with a passionately-held belief in the importance of the issue:

'It definitely has. I have taken it upon myself to give advice and sometimes people come to me when making a story on suicide. I think it is important to let journalists know that you can make a good story and still be ethically correct. I feel obligated to contest the work of other journalists when I see some of the bad work that is being made. There will always be new reporters who don't know the rules of the WHO, and old reporters who have forgotten them and it is extremely important to make them read and think about the rules, so we can have better journalism on suicide in Denmark.'

### Other impacts of awards

A number of the Australian winners commented on the award's value in terms of individual recognition for their good work practice, and in terms of an acknowledgement that journalists and producers care about issues regarding responsible reporting of suicide. They felt that the latter was particularly important because the media are often criticized for poor reporting, and the award counters this by recognizing the positive role that media professionals can play in this area. They also commented that the award ceremony itself was a positive opportunity to discuss suicide-related issues in a responsible fashion, not only with other journalists but also with a range of individuals within, and interested in, the suicide prevention and mental health sectors.

In Belgium, the awards were also viewed very positively by recipients. Like the Australian winners, they expressed pride at being recognized for the quality of their work. They perceived the award as a positive, proactive initiative which emphasized good examples of media reporting, rather than criticising bad examples. As one recipient put it:

'It's a very nice way of working. The tendency to react in a negative way gets countered. This initiative works in a positive manner, it stimulates to do good.'

The Danish recipients also universally reported that winning the award was an important recognition of their work, and that the awards were a positive way of addressing how the media can best report sensitive issues regarding suicide.

## Discussion

This study was small in scale, but captured the views of all but one of past recipients of awards for responsible reporting of suicide in three countries. Clear themes emerged from our interviews with these recipients, and although there were there were some differences in responses both within and across countries, the similarities outweighed these differences. In qualitative research terms, it is fair to say that 'saturation' was reached.

Our findings suggest that media awards show promise as a method of reinforcing national and international media guidelines on reporting suicide. The recipients of awards in our three countries were proud to have had their achievements recognized in this way, and had developed a heightened awareness of the issues inherent in reporting suicide. Although relatively few had prepared subsequent stories on suicide, a number had been given opportunities to provide advice to other media professionals about how best to approach this sensitive topic. Recipients viewed the awards as an important means by which good quality reporting can be rewarded, and a springboard for raising community awareness about suicide.

To fully capitalise on the potential of media awards for improving reporting of suicide, the awards need to become more prominent. Several of the award recipients in each country indicated that they were not aware of the existence of the awards until they were nominated for and won one. In Australia, Belgium and Denmark the awards are driven by the suicide prevention sector, rather than the media industry. Greater buy-in from the latter is necessary to raise the profile of the awards, so that they become as prestigious and coveted as other media industry awards.

There may also be opportunities to 'value-add' to the awards. Currently, they are 'stand-alone' entities. Once the award is given, the winner can 'put it on the trophy shelf' and continue his or her regular reporting responsibilities. Although the winners we interviewed tended not to do this, and continued to take an interest in suicide prevention in general and reporting of suicide in particular, there was no onus on them to do so. Consideration might be given to ways in which future awards might encourage recipients to promote guidelines on responsible reporting and provide mentorship to colleagues. This is important, because all media professionals who report suicide-related issues are the primary target group for the awards, not just the award recipients.

It is also crucial that the guidelines which the awards complement are widely available and well respected by the media industry. Australia is regarded as a leader in this regard. Its strategic dissemination strategy, led by the Hunter Institute of Mental Health, has resulted in a high proportion of the media industry being exposed to the guidelines [14]. Other countries have not yet experienced the same degree of success. Widespread promotion of relevant guidelines - e.g., by offering training to journalists and other media professionals - is necessary to underpin the awards. In turn, the awards can increase the likelihood that media professionals will embrace the guidelines.

The experience from Australia, Belgium and Denmark suggests that media awards which recognize responsible reporting of suicide are extremely worthwhile. Ongoing support for these awards will be crucial if they are to fulfill their potential in helping to combat the Werther effect.

## Competing interests

The authors declare that they have no competing interests.

## Authors' contributions

JP, KA and MN devised the original idea for the study and AD assisted with refining the study design. AD, AH, KA and MM conducted the interviews. AD conducted the data analysis and took the lead on writing the paper. JP, KA, MN, MM and AH provided input on successive drafts of the paper. All authors read and approved the final manuscript.
